# Aetiology of viral hepatitis among jaundiced patients presenting to a tertiary hospital in Ghana

**DOI:** 10.1371/journal.pone.0203699

**Published:** 2018-09-12

**Authors:** Michael Owusu, Joseph Kofi Bonney, Augustina Angelina Annan, Gifty Mawuli, Kennedy Okyere, Mohamed Mutocheluh, Juliana Aryeequaye, Nicholas Kwabena Adjei, Mary Afihene, Kathryn Spangenberg, Justice Sylverken, Ellis Owusu-Dabo, Christian Drosten, Yaw Adu-Sarkodie

**Affiliations:** 1 Kumasi Centre for Collaborative Research in Tropical Medicine, Kwame Nkrumah University of Science and Technology, Kumasi, Ghana; 2 Department of Virology, Noguchi Memorial Institute for Medical Research, University of Ghana, Legon, Ghana; 3 Department of Theoretical and Applied Biology, Kwame Nkrumah University of Science and Technology, Kumasi, Ghana; 4 Department of Clinical Microbiology, School of Medical Sciences, Kwame Nkrumah University of Science and Technology, Kumasi, Ghana; 5 Department of Child Health, Komfo Anokye Teaching Hospital, Kumasi, Ghana; 6 Department of Medicine, Komfo Anokye Teaching Hospital, Kumasi, Ghana; 7 Department of Family Medicine, Komfo Anokye Teaching Hospital, Kumasi, Ghana; 8 Department of Global Health, School of Public Health,Kwame Nkrumah University of Science and Technology, Kumasi, Ghana; 9 Institute of Virology, Charité - Universitätsmedizin Berlin, Berlin, Germnany; Kaohsiung Medical University, TAIWAN

## Abstract

**Background:**

Viral hepatitis continues to play significant role in causing morbidity and mortality in sub-Saharan Africa. Apart from the few population based studies available, not many have investigated the burden of these viruses in jaundiced patients. Among the few studies, hepatitis E is the least studied among jaundiced patients. This study was aimed at describing the frequency, distribution and risk of the different hepatitis viruses among jaundiced patients reporting to the second largest teaching hospital in Ghana.

**Methods:**

From November, 2015 to April, 2016, a cross-sectional study was conducted among jaundiced patients attending the Komfo Anokye Teaching Hospital. Between 3–5 ml of blood was collected from each patient and screened for viral hepatitis agents using both serologic and molecular-based assays.

**Results:**

In the 155 patients recruited, hepatitis B was the most prevalent [54.2% (95% CI = 46.0%–62.2%)] followed by hepatitis E [32.9% (95% CI = 25.6–40.9%)]. Most cases of hepatitis E occurred as co-infections with hepatitis B (18%), with the predominant clinical feature being hepatocellular carcinoma. Risk factor variable analysis showed middle and older aged individuals were more at risk of hepatitis B exposure whereas younger age groups (<18 years) were more at risk of hepatitis E virus infection.

**Conclusion:**

Hepatitis viruses are still important in the viral aetiology of jaundice in Ghana. Hepatitis B and hepatitis E co-infections could play significant roles in causing severe disease. A more aggressive approach needs to be adopted in order to reduce the morbidity and mortality associated with hepatitis causing viruses in Ghana and other developing countries.

## Introduction

Viral hepatitis continues to pose significant public health challenges in developing countries especially in sub-Saharan Africa. Five main causes of viral hepatitis have been identified to date. These are hepatitis A (caused by hepatitis a virus; HAV), hepatitis B (HBV), hepatitis C (HCV), hepatitis D (HDV) and hepatitis E (HEV).

HBV and HCV are transmitted mainly through body fluids. HBV is the most common hepatitis causing virus accounting for nearly 2 billion infections globally and around 5–10% of chronic infections among adult populations in sub-Saharan Africa and East Asia [1, 2]. Similarly, an estimated proportion of 2.8% of the world’s population representing 180 million individuals are infected with HCV [3]. In Ghana, HBV and HCV respectively account for about 10–15% and 3% of infections in the general population [4, 5]. These two viruses have been implicated in the development of hepatocellular carcinoma (HCC). Whereas about 1–5% of chronic HCV sufferers are likely to develop HCC, up to 50% of HCC cases are attributed to both direct and indirect oncogenic effects of HBV [6, 7]. HAV and HEV on the other hand are transmitted through the faecal-oral route and often associated with asymptomatic illness but can cause acute disease characterised by clinical jaundice. HEV especially may develop into fulminant hepatitis with a case fatality rate between 1 and 2% in the general population [8]. This fatality can rise to over 40% in pregnant women [9, 10]. Few cases of sporadic outbreaks of HAV and HEV have been reported in Ghana, Kenya, and other developing countries [11–13]. A report by the World Health Organisation (WHO) and other researchers show that more than 1.5 million cases of hepatitis A and 20 million cases of HEV infections occur annually resulting in over 3 million symptomatic cases [14, 15].

Infections associated with HAV and HEV still remain a public health concern in Africa because of poor sanitation, unhygienic practices and lack of access to clean water. Individuals in rural parts of Africa who use wells are reported to have 8.6% case fatality whereas those relying on river and pond water have 2.5% and 0.8% case fatality rates, respectively [16]. Of the limited data that exist on the prevalence of hepatitis causing viruses in Ghana and other parts of Africa, a higher proportion were extracted from healthy populations. Information on the occurrence of these viruses in patients with hepatic related jaundice is limited. Knowledge on the burden of these viruses in jaundiced patients would provide a good estimate of the morbidity and mortality associated with exposure to hepatitis viruses and help direct therapies.

This study was conducted to determine the epidemiology of hepatitis causing viruses among patients presenting with jaundice to the Komfo Anokye Teaching Hospital (KATH).

## Methods

### Ethics and consent to participate

This study was approved by the Committee on Human Research Publication and Ethics of the Kwame Nkrumah University of Science and Technology. Informed consent was obtained from all patients prior to recruitment. Consent to publish the findings of this study was also obtained from all patients during the consenting process. It was however made known to them that their names would be excluded from the manuscript.

### Study area

The study was carried out at KATH; the second largest tertiary medical facility in Ghana. KATH is approximately a 1200 bed tertiary medical facility located in Kumasi; the Ashanti region of Ghana. The hospital serves over 4 million people within its catchment area. The average patient utilisation is approximately 350,000 [17]. Three units in the hospital attend to patients presenting with jaundice. These are the Medicine Gastroenterology, Paediatric Gastroenterology and the Family Medicine units. The Medicine and Child Health units respectively identified liver disease and jaundice as the second and third causes of death in the 2015 annual year [18].

### Study design and sample size

We conducted a cross-sectional study to determine the sero- and molecular epidemiology of hepatitis viruses among jaundiced patients reporting to the hospital from November, 2015 –April, 2016. Recruitments were done from the Medicine, Child Health and the Family Medicine units of the hospital. Patients with ages 3 months and above were assessed by specialist physicians and those found to be jaundiced were prospectively enrolled. Prior to the study, a sample size of at least 153 was determined taking into consideration a two sided alpha level of 5%, a study power of 80% and design effect of 1. The estimate was based on a HBsAg seroprevalence of 50.6% among patients presenting with jaundice [19].

### Patient sampling

For all patients, blood samples processed into serum were collected after informed consent or assent was sought. Variables including socio-demographic characteristics, history of presenting medical complains, exposure to livestock, consumption of pork and serum liver markers were entered on standard questionnaires. Attempts were made to sample every subject that presented to the clinics within the study period in order to reduce bias that may result from sampling. All samples were transported to the Kumasi Centre for Collaborative Research in Tropical Medicine under cold chain conditions for processing and testing.

### Laboratory processes

All specimens collected were screened for HBV, HCV and HEV with both serologic and molecular tools. HAV was screened using only molecular methods. Prior to molecular analysis, nucleic acids of all serum samples were extracted and purified with the Viral RNA Mini kit (Qiagen, Hilden, Germany). Extraction was done following the manufacturer’s protocol.

#### Hepatitis A virus

Molecular test for HAV was performed using a OneStep Reverse Transcription (RT)-PCR reagent kit (Qiagen, Hilden, Germany). Briefly, a 25 μl reaction mix contained 1 μl of 10 mM dNTPs, 1 μl of 10 μM each of forward and reverse primers, 5 μl of Q solution, 6 μl of RNase free water, 1 μl of enzyme mix and 5 μl of nucleic acid extract. Cycling conditions comprised RT at 48°C for 30 minutes, denaturation of 94°C for 2 minutes and 40 cycles of 95°C for 15 seconds, 50°C for 30 seconds and 68°C for 60 seconds. PCR products were visualized on a 2% agarose gel with 2 μl of Ethidium bromide per 100 ml gel solution, and bands of 510 bp were noted as positive for HAV. For all the tests, known control samples positive for HAV were used to validate the run.

#### Hepatitis B

HBV testing was done using both molecular and serological methods. Molecular tests were done using real time PCR. Briefly, a 25μl reaction mix contained 0.88 μl of 10mM dNTPs, 1.32μl of 50mM MgCl_2_, 4.4μl of 10X Platinum buffer, 0.88μl of 10 μM each of forward and reverse primers mix (made up of equal volumes of R1, R2, R3), 12.7μl of water, 0.5μl of Platinum Taq and 0.3 μl of viral DNA. Thermocycling parameters comprised of denaturation at 95 °C for 10 min, followed by 3 cycles at 95 °C for 15 seconds and 60°C for 45 seconds and then 45 cycles at 93 °C for 10 seconds and 60°C for 40 seconds.

Serological tests were performed using Abon HBsAg test kit (Biopharm, China)[20]. The test strip is a qualitative immunoassay for identifying HBsAg. The kit principle is based on the serum or plasma antigens binding to the pre-coated anti-HBsAg antibody on the strip. Testing was performed by dipping the strip in serum for 15 seconds.

#### Hepatitis C

HCV testing was similarly performed using both molecular and serological method. Molecular testing was done using One Step RT-PCR Kit (Qiagen, Hilden, Germany). Briefly, a 25μl reaction mix contained 5μl of 5x Reaction Buffer, 5 μl of Q Solution, 1μl of 10mM dNTPs, 1.5μl of 10μM each of forward and reverse primers, 1μl of enzyme mix and 5μl of RNA extract. Cycling conditions comprised of an RT step of 50°C for 30 minutes, denaturation of 95°C for 15 seconds and 45 cycles of 95°C for 15 seconds, 60°C for 15 seconds and 72°C for 30 seconds. DNA products were visualized on a 2% agarose gel and band sizes equivalent to 380bp were noted as HCV viruses. Control strains of known HCV viruses were used to validate PCR testing.

For rapid serologic tests, Abon (Biopharm, China)[20] HCV test strip was used. The kit is a qualitative immunoassay for identification of antibody to HCV. The membrane within test strip is pre-coated with HCV antigen. During the test, the sample antibodies bind to the pre-coated HCV antigen resulting in a colored line. The test was performed by dipping the strip in serum specimen for 15 minutes. Samples with coloured lines at both the test and control windows were noted as positive for HCV.

#### Hepatitis E

Serum samples were screened for HEV IgG and IgM class antibodies with AXIOM ELISA assays (Axiom diagnostics, Germany) and real time PCR using Taqman based probes. For the indirect ELISAs, 100μl of sample diluent was first added to microplates already pre-coated with recombinant antigens corresponding to the structural regions of HEV Open Reading Frame-2. Ten microliters (10μl) of study samples including negative and positive controls were added to each pre-labelled sample wells. The plates were incubated at 37°C and 100μl of horseradish peroxidase (HRP) followed by the addition of 50μl of chromogen A and B substrates. The final reaction was read at 450nm. The cut off values (C.O) for the wells (samples) were calculated according to the manufacturer’s directions using the formula: C.O = *Nc + 0.16, (*Nc = the mean absorbance value for three negative controls).

Real time RT-PCR was performed on the extracted nucleic acid to identify the ORF3 of HEV virus. The reactions were carried out in a 25μl mixture containing 2μl of 10 μM each of forward and reverse primer, 1μl of each dNTPs and bovine serum albumin, 5μl of 5X OS buffer, 7.3μl of RNASE free water, 1μl of enzyme mix and 5μl of nucleic acid extract. Thermocycling parameters comprised of reverse transcription at 50°C for 30 min, denaturation at 95°C for 15min, followed by 45 cycles at 95°C for 15 seconds and annealing at 58°C for 30 seconds. The sequences of probes and primers are shown in Table 1.

**Table 1:** List of primers and target regions of sequences.

Virus	Reagents	Primers sequences (5’–3’)	Target region	Amplicon length	Reference
**HAV**	OneStep RT-PCR Kit (Qiagen)	2870 (GACAGATTCTACATTTGGATTGG), 3381 (CCATTTCAAGAGTCCACACACT)	VP1/2A junction	510	[21]
**HBV**	Platinum Taq polymerase (Invitrogen)	F- GAT GAG GCA TAG CAG CAG GAT R1- CAA CCT CTT GTC CTC CAA CTT GT R2- AAC CTC CTG TCC TCC AAC TTG T R3- CAA CCT GTT GTC CTC CAA TTT GT P—FAM-ATC GCT GGA TGT GTC TGC GGC GTT-TAMRA	PreS2 gene	N/A	[22]
**HCV**	OneStep RT-PCR Kit (Qiagen)	Pr3 (TATGAYACCCGCTGYTTTGACTC), Pr4 (GCNGARTAYCTVGTCATAGCCTC)	NS5B	380	[23]
**HEV**	OneStep RT-PCR Kit (Qiagen)	HEV_F GGTGGTTTCTGGGGTGAC HEV_R AGGGGTTGGTTGGRTGRA HEV_P FAM-TGATTCTCAGCCCTTCGC-MGB	ORF3	N/A	[24]

### Ethical approval

The study protocol was approved by the Committee for Human Research, Publications and Ethics (CHRPE) of KATH and School of Medical Sciences, KNUST, Kumasi, Ashanti region, Ghana. Prior to recruiting the patients, the study protocol was explained to them in their local dialect. Written informed consent was obtained for data and sample collection from adults and parents or guardians of children. For children between 13 and 17 years, assent was also obtained.

### Statistical analysis

All data obtained from patients were recorded using Microsoft Excel. Subsequent analysis was performed using R statistical software (version 3.3.2). Categorical variables and their association with hepatitis viruses were analysed with Fisher’s exact or Chi-square test where appropriate. In defining positivity of HBV viruses, a combined binary variable was created from both PCR and serology results to allow for correlation with clinical and socio-demographic characteristics. All continuous variables were expressed as medians with their inter-quartile ranges (IQR). A non-parametric K-sample test on equality of medians was used to evaluate the differences in the medians of the various subgroups of the continuous variables. Independent risk factors for exposure to various hepatitis viruses were determined by entering possible risk factors which were significant at p < 0.2 into a multivariable logistic regression using both forward and backward selection approaches. Independent risk factors were expressed as the adjusted odd ratios (OR) and 95% confidence interval (CI).

## Results

### Socio-demographic and clinical characteristics of patients

**Fig 1:**
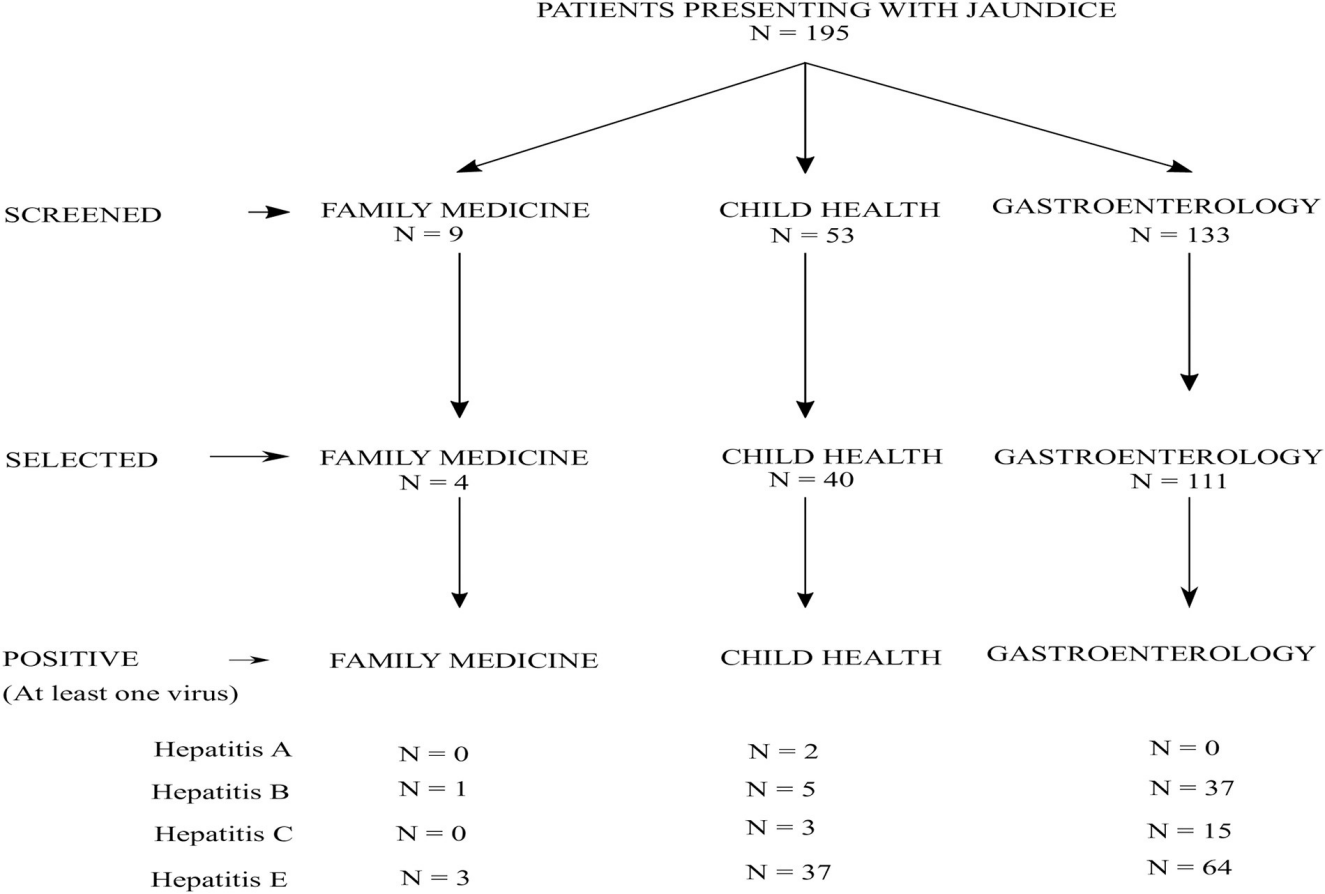
Flowchart of subject recruitment.

Within the period of this study, we screened a total of 195 all jaundiced cases. Fifty three (53) were from the Department of Child Health, 133 from the Department of Gastroenterology and 9 from the Department of Family Medicine. Of the number screened, 155 patients were recruited into this study. The algorithm for screening and recruitment of subjects is shown in Fig 1.

Nine patients were below 12 months of age and 146 patients were above 12 months. The median age of patients below 12 months was 6 months (IQR = 5–7) and that of patients above 12 months was 35 years (IQR = 17–47). Most of the patients were males (92; 59.4%) with the predominant religion being christian (118; 77.1%). Of the 155 patients recruited, 35 (24.3%) had no formal education, 29 (20.1%) had senior high school education, 25 (17.4%) had junior high school education, 16 (11.1%), 16 (11.1%) had tertiary and the rest had both primary and nursery education. Of all patients recruited, 85 (57.8%) were employed.

The study also observed 60 (38.7%) patients presenting with hepatomegaly and 16 (10.3%) presenting with splenomegaly. The predominant diagnosis identified among study subjects was chronic liver disease. Tables 2 and 3 describe summaries of the socio-demographic and clinical characteristics of study patients.

**Table 2:** Socio-demographic characteristics of patients.

Variable	Frequency	Percent
**Age**		
3 months– 18yrs	47	30.3
19yrs– 40yrs	59	38.1
41yrs– 79yrs	49	31.6
**Gender**		
Male	63	40.6
Female	92	59.4
**Religion**		
Christian	118	77.1
Muslim	31	2.6
Others	4	20.3
**Highest Level of Education**		
Nursery	12	8.3
Primary	27	18.8
Junior High School	25	17.4
Senior High School	29	20.1
Tertiary	16	11.1
No formal Education	35	24.3
**Occupational Status**		
Employed	85	57.8
Unemployed	62	42.2
**Source of Drinking Water**		
Mostly Borehole	20	14.3
Mostly Pipe water	61	43.6
Mostly Sachet water	42	30
Others	17	12.1
**Rearing of livestock**		
Rear livestock	52	36.9
Don’t rear livestock	89	63.1
**Pork Consumption**		
Consume Pork	53	37.1
Don’t consume Pork	90	62.9
**History of Blood Transfusion**		
No	95	67.4
Yes	46	32.6
**History of Surgery**		
No	120	85.7
Yes	20	14.3

**Table 3:** Clinical characteristics of subjects.

Variable	Summary
Total	155
**Hepatomegaly**	
No	95 (61.3)
Yes	60 (38.7)
**Splenomegaly**	
No	139 (89.7)
Yes	16 (10.3)
**Diagnosis**	
Acute liver disease	10 (7.2)
chronic liver disease	59 (42.8)
hepatic encephalopathy	9 (6.5)
Hepatocellular carcinoma	32 (23.2)
Liver cirrhosis	16 (11.6)
Others	12 (8.7)
**Upper Gastrointestinal Bleeding**	
No	134 (86.5)
Yes	21 (13.5)
**Respiratory rate**	
mean(SD)	28.1 (12.1)
**Heart rate**	
mean(SD)	100.4 (22.8)
**Clinical outcome**	
Died	5 (3.2)
Discharged	150 (96.8)

### Virus distribution

The present study identified four hepatitis viruses among the study patients. Of the 155 subjects, 108 (70%) had one or more virus exposures. HBV was the most prevalent virus with detectable viral DNA in 43 (27.7%) patients and HBsAg detection in 70 (45%). The overall prevalence of HBV was [54.2% (95% CI = 46.0%–62.2%)]. HAV RNA was identified in 2 patients (1.3%); one was identified in a 9-year-old female and the other in a 2-year old male. All samples were negative for HCV and HEV viral RNA.

Based on serologic methods, the prevalence of HCV antibodies was 11.6% (95%CI = 7.0%–17.7%). HEV IgM antibodies were identified in 3 (1.9%) patients and IgG antibody was identified in 51 (32.9%) patients. The overall prevalence of HEV infection was 32.9% (95%CI = 25.6–40.9%).

Our study also identified cases of single and multiple infections. Single infections occurred in 48 patients (31%) for HBV, followed by 11 patients (7.1%) for HEV and 5 patients (3.2%) for HCV. Table 4 summarizes combinations of infections observed.

**Table 4:** Frequency table of mono and co-infections.

		Frequency	Percentage	Cumulative Percentage
***Mono-infections***	Only HAV	1	0.6	0.6
	Only HBV	48	31.0	31.6
	Only HCV	5	3.2	34.8
	Only HEV	11	7.1	41.9
***Dual Infections***	HAV + HBV	1	0.6	42.6
	HBV + HEV	29	18.7	61.3
	HBV + HCV	2	1.3	62.6
	HCV + HEV	7	4.5	67.1
***Triple Infection***	HBV + HCV + HEV	4	2.6	69.7
***All Negative***	Negative for all viruses	47	30.3	100.0
	Total	155	100	100

### Viruses stratified by clinical and socio-demographic characteristics

A stratification of infections by socio-demographic characteristics showed that age was significantly associated with HBV and HEV but not HCV infection. Interestingly, being employed (as opposed to unemployed) was significantly associated with HBV and HEV infection. Similarly, there was significant association between HEV exposure and christian religion. No association was found between HEV exposure and rearing of livestock, consumption of pork or drinking from specific water sources. Similarly, for HCV, there was no association between any of the demographic variables and HCV infection. Table 5 shows the association between the three commonest viruses and the socio-demographic variables.

**Table 5:** Socio-demographic characteristics of study subjects stratified by hepatitis viruses.

	Hepatitis B Positive	Hepatitis C Positive	Hepatitis E Positive
Total	84	18	51
**Age of Subjects (years**)	*p-value < 0*.*001*	*p-value = 0*.*314*	*p-value (<0*.*001)*
< 18	11 (13.1)	3 (16.7)	4 (7.8)
19–40	43 (51.2)	7 (38.9)	17 (33.3)
41–79	30 (35.7)	8 (44.4)	30 (58.8)
**Gender**	*p-value = 0*.*064*	*p-value = 0*.*925*	*p-value = 0*.*438*
Female	28 (33.3)	8 (44.4)	18 (35.3)
Male	56 (66.7)	10 (55.6)	33 (64.7)
**Religion**	*p-value = 0*.*737*	*p-value = 0*.*444*	*p-value = 0*.*032*
Christian	62 (75.6)	13 (72.2)	33 (64.7)
Others	3 (3.7)	1 (5.6)	2 (3.9)
Muslim	17 (20.7)	4 (22.2)	16 (31.4)
**Level of Education**	*p-value < 0*.*001*	*p-value = 0*.*286*	*p-value = 0*.*139*
No Formal Education	13 (17.3)	5 (35.7)	13 (28.3)
Nursery	2 (2.7)	1 (7.1)	0 (0)
Primary	12 (16)	3 (21.4)	9 (19.6)
JHS	20 (26.7)	0 (0)	6 (13)
SHS	15 (20)	2 (14.3)	11 (23.9)
Tertiary	13 (17.3)	3 (21.4)	7 (15.2)
**Occupational status**	*p-value = 0*.*005*	*p-value = 0*.*648*	*p-value = 0*.*005*
Employed	54 (69.2)	10 (66.7)	38 (79.2)
Unemployed	24 (30.8)	5 (33.3)	10 (20.8)

We also compared the distribution of clinical parameters of patients to three hepatitis viruses. Most patients infected with HBV seem to present mostly with chronic liver disease (40.3%) and hepatocellular carcinoma (31.2%) as compared to those not infected with hepatitis B (p = 0.006). However, among hepatocellular carcinoma patients, single HEV infection was identified in two patients and co-infections of hepatitis B and hepatitis E was identified in 9 patients. A comparison of the liver and renal function tests for hepatitis B and C infected patients to uninfected patients did not show any significant statistical difference (table not shown). For patients infected with HEV, the average creatinine level was higher (median = 71 μmol/l; IQR = 51–100.8) than those not infected (p = 0.023). Of the 155 patients, 5 died. Of the 5, 1 was infected with HEV and three were infected with HBV infection.

To determine the independent risk factors associated with exposure to hepatitis viruses, all variables with p-value below 0.2 were entered into three logistic regression models each for hepatitis B, C and E virus exposures. Age and level of education were identified to be independent risk factors associated with hepatitis B and E infections. No significant factors were associated with hepatitis C virus exposures. Table 6 describes the independent risk factors associated with hepatitis viruses.

**Table 6:** Independent risk factors of hepatitis virus exposure.

Virus	Risk factor	Crude OR (95%CI)	Adj OR (95%CI)	P- value
***Hepatitis B***	Age Groups: Ref = (0–18 years)			
	19–40	9.87 (3.73,26.14)	7.79 (2.27,26.69)	0.001
	41–79	5.73 (2.18,15.08)	8.34 (2.34,29.75)	0.001
	Level of Education: Ref = SHS			
	Tertiary	3.73 (0.87,16.07)	3.6 (0.81,16.12)	0.093
	Nursery	0.21 (0.04,1.13)	1.2 (0.16,9.1)	0.859
	JHS	5.6 (1.35,23.23)	8.97 (1.81,44.33)	0.007
	No formal education	0.47 (0.16,1.34)	0.89 (0.29,2.8)	0.848
	Primary	0.86 (0.3,2.51)	2.92 (0.75,11.43)	0.124
***Hepatitis E***	Age Groups: Ref = (0–18 years)			
	19–40	N/A	0.33 (0.1,1.12)	0.075
	41–79	N/A	0.06 (0.02,0.21)	<0.001

## Discussion

Viral hepatitis is a serious public health problem that continues to affect a majority of individuals living in sub-Saharan Africa. Epidemiological studies in diverse countries have reported approximately 5–10% of adult populations as having chronic liver disease as a result of hepatitis viruses. Although there are some reports on the epidemiology of hepatitis viruses in Ghana and other West African countries, most have focussed on blood donor populations and very few have described the distribution of these viruses among patients presenting with clinical jaundice. Knowledge of viruses in jaundiced patients gives a good estimate of the morbidity and mortality associated with exposure to hepatitis viruses.

The present study identified approximately 70% of all jaundiced patients as having at least one type of hepatitis virus infections with the predominant being HBV (54.2%). Varied proportions of jaundiced patients having hepatitis viruses has been reported in literature[25, 26]. Percentages ranging from 29% [25] up 49% [26] has been reported depending on the type of virus. Our results is not different from a related study conducted in Uganda among patients with acute jaundice syndrome[26]. The reported prevalence in our study is however higher compared to the estimates reported in the general population. A general HBV prevalence of 12.3% has been documented by Ofori-Asenso and Agyeman in a systematic review recently conducted in Ghana [5]. The pooled prevalence was however extracted from studies mostly conducted among healthy populations. Our results present a credible estimate of the burden of HBV associated jaundice in a tertiary hospital and may indeed reflect the population of people living with hepatitis related infections in Ghana. This finding highlights the need for a more aggressive implementation of national policies targeted at reducing the transmission routes of HBV infection. Interventions including childhood and adult vaccination, accurate screening of donated blood, condom use and avoiding multiple sexual partners could all help reduce the burden of HBV in Ghana. Our results also show that most cases of HBV (32.1%) were identified in HCC patients. High incidence of HCC has been reported in the West African sub-region with incidence ranging from 30-50/100,000 population [27]. A review of autopsy records in Ghana showed that liver cancer is the highest cause of mortality among males and the fourth cause of mortality among females [28]. This calls for a more proactive approach in dealing with hepatitis B challenges in Ghana.

Similar to HBV, HCV was the next most important blood borne virus identified in our study cohort with a sero-prevalence of 11.6%. Our estimate is higher than the 5.6% reported in a systematic review conducted in Ghana [29]. The difference was because most reviews and reported estimates were from healthy individuals in the population as compared to our study which was conducted among jaundiced patients. Both HBV and HCC have a common route of transmission hence similar recommendations made about HBV virus exposures will need to be applied to HCV as well. Well-structured national policies are needed to address the high occurrence of these viruses in Ghana.

Our study also investigated the occurrence of two oro-faecal transmitted viruses often associated with jaundice and liver related conditions. Few reports exist on the involvement of oro-faecal transmission route viruses such as hepatitis E in the aetiology of clinical jaundice. Hepatitis E was the most common oro-faecal transmitted virus identified among our study patients with IgG prevalence of 32.9% and IgM prevalence of 1.9%. The detection of acute cases of HEV (IgM positive) and a high prevalence of IgG together indicate the presence and possible circulation of the virus in the Ghanaian population. Our estimate falls within the prevalence range of 5.8%–71.5% previously reported by Ofori-Asenso et al. [30]. Although most cases of HEV infections are thought to be self-limiting, severe cases could also occur [13, 31]. Nine (9) of the 51 HEV cases occurred as HBV co-infections in patients with hepatocellular carcinomas which suggests a higher risk for HEV infection among individuals with hepatitis B.

One of the 5 patients who died was positive for anti-hepatitis E IgM antibodies. Attempt to isolate the virus RNA and characterise the strain proved futile. Similar observations have been reported where HBV superinfection with hepatitis E infections could lead to clinical progression of liver disease [31]. A plausible explanation is that the patients infected with HBV may show an altered immune response and thus become more susceptible to secondary infection with HEV. The high prevalence of HEV could either be due to the poor hygienic conditions which may facilitate oro-faecal transmission of the virus or having close contact with livestock (especially swine) known to carry the virus. Eating of under-cooked meat from pigs could also serve a route of virus transmission since pigs are probable reservoirs of the virus. Close contact with swine has been associated with 9.1 odds of exposure to HEV [32]. Our study did not however identify any association between hepatitis E exposure and rearing livestock or consuming pork. However, we found a significantly positive correlation with Christian versus Muslim religion, which provides a hint toward a possible association with pork consumption.

We also identified 2 cases of HAV among our study patients. The two cases were identified in children less than 10 years; both having chronic liver disease with one occurring as a co-infection with HBV and the other as a single infection. As established with HEV, HAV is also self-limiting with less potential to cause liver damage. Minor cases could however be associated with severe ailments as shown in our present study. Ghana and most developing countries are either not implementing national policies on management and treatment options for HAV infections widely enough or these policies are non-existent. Like hepatitis E, the virus is transmitted through poor hygienic practices and dependence on contaminated water. The identification of these viruses in children with chronic liver disease emphasizes the need for more surveillance and advocacy for interventions that will reduce the burden of the disease.

The present study observed employment and older age as important factors associated with hepatitis B and E infection. A cross-tabulation of age by employment showed that most unemployed subjects were actually young students and children whereas those employed were older subjects and hence mostly working. It was therefore not surprising to find low occurrence of hepatitis B among younger or unemployed compared to older or employed subjects because most of them had been part of Ghana’s expanded programme on immunisation which sought to vaccinate newborns against hepatitis B. Similar observations has been reported elsewhere[33, 34].

Our study comes with some limitations including ruling out other possible viral agents (yellow fever virus and other viral haemorrhagic fever viruses) that may contribute to liver inflammation with the subsequent development of jaundice Additionally, lack of antibody testing for HAV and the small sample size could underestimate our results. However, these limitations do not invalidate our findings but emphasize the need for larger studies to fully describe the epidemiology of these viruses in Ghana and other developing countries.

## Conclusion

Our study has brought to fore the importance of hepatitis viruses causing various forms of hepatitis related conditions among jaundiced patients. Most importantly we have shown that hepatitis E is quite prevalent among jaundiced patients and could occur often as co-infections with other hepatitis viruses. With the increase in urbanisation which goes with poor sanitation and certain lifestyle that fuel sexual transmission, there is a need for a more aggressive approach to be adopted in order to reduce the morbidity and mortality associated with hepatitis causing viruses in Ghana and other developing countries.

## Supporting information

S1 Table STROBE statement. (DOC)

## Abbreviations

CHRPE: Committee for Human Research, Publications and Ethics
CI: Confidence interval
HAV: Hepatitis A virus
HBV: Hepatitis B virus
HCC: Hepatocellular carcinoma
HCV: Hepatitis C
HDV: Hepatitis D
HEV: Hepatitis E
HRP: Horseradish peroxidase
IQR: Inter-quartile ranges
KATH: Komfo Anokye Teaching Hospital
OR: Odd ratios
RNA: Ribonucleic Acid
WHO: World Health Organisation
